# Effects of Selenomethionine on Cell Viability, Selenoprotein Expression and Antioxidant Function in Porcine Mammary Epithelial Cells

**DOI:** 10.3389/fnut.2021.665855

**Published:** 2021-07-26

**Authors:** Jun Chen, Yinzhi Zhang, Yantao Lv, Min Tian, Jinming You, Fang Chen, Shihai Zhang, Wutai Guan

**Affiliations:** ^1^Guangdong Provincial Key Laboratory of Animal Nutrition Control, College of Animal Science, South China Agricultural University, Guangzhou, China; ^2^Jiangxi Province Key Laboratory of Animal Nutrition, Engineering Research Center of Feed Development, Jiangxi Agricultural University, Nanchang, China; ^3^College of Animal Science and National Engineering Research Center for Breeding Swine Industry, South China Agricultural University, Guangzhou, China

**Keywords:** antioxidant, cell viability, porcine mammary epithelial cells, selenomethionine, selenoproteins

## Abstract

This study investigated the effects of selenomethionine (Se-Met) on the cell viability, selenoprotein expression, and antioxidant function of porcine mammary epithelial cells (pMECs) to reveal the underlying molecular mechanism of Se-Met on the lactation performance and antioxidant capacity of sows *in vitro*. The pMECs were used as an *in vitro* model and were treated with various concentrations of Se-Met (0, 0.5, 1, 2, and 4 μM). Cells were analyzed for cell viability, selenoprotein transcriptome, selenoprotein expression, and antioxidant enzyme activities. The results showed that, with increasing Se-Met concentrations, cell viability first increased and then decreased at 24, 48, or 72 h posttreatment with maximum values at 0.5-μM Se-Met. As the Se-Met concentrations increased, the mRNA expression of 17 selenoproteins first upregulated and then downregulated, with maximum values at 0.5-μM Se-Met. The 17 selenoproteins included *SEPHS2, SELENOP, GPX1, GPX2, GPX3, GPX6, TXNRD1, SELENOK, SELENOW, DIO1, DIO2, DIO3, SELENOF, SELENOS, SELENOH, SELENOI*, and *SELENOT*. Additionally, the protein expression levels of SEPHS2, SELENOP, GPX1, and TXNRD1 and the activities of glutathione peroxidase and thioredoxin were highest at 0.5-μM Se-Met. In conclusion, 0.5-μM Se-Met promotes cell viability partially by improving selenoprotein expression and antioxidant function in pMECs, which provides evidence for the potential ability of Se-Met to improve mammary gland health in sows.

## Introduction

Lactating sows have a significant demand for energy and nutrients due to a substantial metabolism, and the mammary gland is one of the most metabolically active tissues in lactating sows. It has been reported that sows produce 60-g milk/kg body weight, which is even higher than that of dairy cows (50-g milk/kg body weight) (1). Once sows enter the lactating stage, mammary gland metabolism rapidly increases, and genes involved in the synthesis of milk components (protein, fat, lactose, etc.) are significantly upregulated in mammary gland tissues, such as *CSN1S2, LALBA, WAP, SAA2*, and *BTN1A1*, and the transcriptional regulators *SREBP1* and *XBP*1 are activated (2). Compared with non-lactating sows, there are 632 differentially expressed genes in the liver of lactating sows, which are mainly involved in the metabolism of threonine, serine, glycine, glutathione, pyruvate, fatty acids, and glycerophospholipids, PPAR signaling, focal adhesions, and the citric acid cycle (3), and the genes associated with the synthesis and uptake of carnitine in the liver are also significantly upregulated (4). However, substantial metabolism leads to a large amount of oxygen consumption, which is prone to generate many oxygen-free radicals and lipid peroxide. Indeed, Rosenbaum et al. (5) found that lactation activated the Nrf2 pathway in the sow liver, which is a stress signaling pathway associated with inflammation and oxidative stress.

It has been demonstrated that selenium (Se) promotes the development of the mammalian mammary gland (6) and affects milk production (7–9), and milk composition in dairy livestock (10–13), as well as the maternal transfer of immunoglobulins *via* milk (13–17). The beneficial effect of Se may be related to the fact that Se can improve antioxidant functions and reduce tissue damage in livestock (18, 19) because lactation is a process involving high metabolism and a large number of free radicals. Currently, 25 selenoproteins have been found in pigs, and at least half of them are associated with antioxidant functions (20). Selenium (Se) plays a crucial role in cell growth, the cell cycle, and apoptosis (21), and it is an essential regulator of the expression and activity of selenoproteins in mammary tissue (22). Se status is one of the most critical factors determining selenoprotein expression (23). The Se is an essential regulator of the expression and activity of selenoproteins in mammary tissue (22). The Se is incorporated into selenoproteins in the form of selenocysteine, and the biological functions of Se are mediated *via* selenoproteins (23). Se phosphate synthase 2 (SEPHS2) plays an essential role in selenoproteins synthesis, and it plays a self-regulating role in selenoproteins synthesis (24). SEPHS2 catalyzes the synthesis of an active Se donor—selenophosphate (25), which is essentially needed for selenocysteine synthesis (24). Glutathione peroxidase (GPX) is a family of antioxidant enzymes that rely on glutathione to reduce peroxide to non-toxic water to protect cells from oxidative damage (26). There are eight GPX subtypes in mammals, of which GPX1, GPX2, GPX3, GPX4, and GPX6 have selenocysteine residues present in their active sites, while GPX5, GPX7, and GPX8 active sites are cysteines in place of selenocysteine (24). Thioredoxin reductase (TXNRD) plays an essential role in mammalian redox signals. Mammals have three TXNRD isozymes (TXNRD1, TXNRD2, and TXNRD3) (21). However, studies on the effects of Se on selenoprotein expression and antioxidant capacity in pMECs have not been reported.

Therefore, this study aimed to investigate the effect of selenomethionine (Se-Met) on selenoprotein expression and antioxidant function in pMECs to reveal the underlying molecular mechanism of Se-Met on the lactation performance and antioxidant capacity of sows *in vitro*.

## Materials and Methods

### Cell Culture

The pMECs used in this study were previously isolated and characterized from the mammary glands of lactating sows in our lab and were used to evaluate the synthesis and/or transport of amino acids, fatty acids, and lactose in sows in our previous studies. Cells were incubated at 37°C in 5% CO_2_. Cells were cultured in a complete medium according to the formula of Jaeger et al. (27), which consisted of Dulbecco's Modified Eagle Medium/Nutrient Mixture F-12 (DMEM/F12, GIBCO), 10% fetal bovine serum (FBS, PAA), 1% antibiotic/antimycotic solution (10,000-U/mL penicillin, 10-mg/mL streptomycin sulfate, 25-μg/mL amphotericin B, GIBCO, I-15240), 10-μg/mL insulin (Sigma, I 6634) and 1-μg/mL hydrocortisone (Sigma-Aldrich). Cell culture media contain approximately 20 nM selenium due to the presence of 10% fetal calf serum (28).

### Cell Viability Assay

Cell viability was assessed using the CCK-8 assay (Dojindo, Japan) according to the instructions of the manufacturer. Briefly, pMECs were seeded into 96-well microplates at 200 μL/well at 2 × 10^4^ cells/mL and cultured in a complete medium at 37°C and 5% CO_2_ for 48 h. Then, the cells were treated with different levels of Se-Met (0, 0.5, 1, 2, or 4 μM). At 24, 48, and 72 h posttreatment, 20-μL CCK-8 was added to each well and incubated for 4 h at 37°C and then measured using a microplate reader at a wavelength of 450 nm.

### RNA Isolation and Quantitative Real-Time PCR

Porcine mammary epithelial cells were seeded into six-well plates at 2 mL/well at 5 × 10^4^ cells/mL and cultured in a complete medium at 37°C and 5% CO_2_ for 48 h. Then, the cells were treated with different levels of Se-Met (0, 0.5, 1, 2, or 4 μM) for 48 h. After that, total RNA was extracted from pMECs using TRIzol (Invitrogen catalog, No. 15596-026) according to the instructions of the manufacturer. The quality and the quantity of RNA were analyzed by an Agilent Bioanalyzer 2100 using an RNA 6000 Labchip kit. Potential DNA contamination in the extraction was eliminated using a DNA-free kit (Ambion, catalog No. AM1906), and the RNA quality was verified by both agarose gel (1%) electrophoresis and spectrometry (A260/A280). First-strand cDNA synthesis was performed by using a PrimeScript RT reagent kit with a gDNA eraser (Takara, Dalian, China). cDNA was synthesized from 1 μg of total RNA using SuperScript III reverse transcriptase according to the instructions of the manufacturer. The mRNA levels of 25 selenoprotein genes were analyzed by qPCR using SYBRR Green PCR Master Mix according to the instructions of the manufacturer (Cat # RR047A, Takara). Primers for the 25 selenoprotein genes (see Table 1) were referenced from the study of Zhao et al. (29), and primers for the β-actin gene (*ACTB*) were from our previous study (30). The 2^−Δ*ΔCt*^ method was used for quantification with the β-actin gene as a reference gene, and the relative abundance was normalized to the control.

**Table 1:** Primers used for RT-PCR^1^.

**Gene**	**Accession number**	**Primer pairs (5^**′**^ to 3^**′**^ direction)**
*DIO1*	AY533206	F: CATGGCCAAGAACCCTCACT
		R: CCAGAAATACTGGGCACTGAAGA
*DIO2*	AY533207	F: CGCTGCATCTGGAAGAGCTT
		R: TGGAATTGGGTGCATCTTCA
*DIO3*	AY533208	F: TGAAGTGGAGCTCAACAGTGATG
		R: TGTCGTCAGACACGCAGATAGG
*GPX1*	AF532927	F: GATGCCACTGCCCTCATGA
		R: TCGAAGTTCCATGCGATGTC
*GPX2*	DQ898282	F: AGAATGTGGCCTCGCTCTGA
		R: GGCATTGCAGCTCGTTGAG
*GPX3*	AY368622	F: TGCACTGCAGGAAGAGTTTGAA
		R: CCGGTTCCTGTTTTCCAAATT
*GPX4*	NM_214407	F: TGAGGCAAGACGGAGGTAAACT
		R: TCCGTAAACCACACTCAGCATATC
*GPX6*	NM_001137607	F: GAGCTGAAGCCTTTTGGTGTAGTT
		R: CTTTGCTGGTTCTTGTTTTCCA
*MSRB1*	EF113597	F: ATCCCTAAAGGCCAAGAATCATC
		R: GGCCACCAAGCAGTGTTCA
*SELENOF*	EF178474	F: ACAGCCCTGCCAAGCAGAT
		R: AACAGGGAGGCTGGGTAACAC
*SELENOH*	HM018602	F: TGGTGGAGGAGCTGAAGAAGTAC
		R: CGTCATAAATGCTCCAACATCAC
*SELENOI*	EST	F: GATGGTGTGGATGGAAAGCAA
		R: GCCATGGTCAAAGAGTTCTCCTA
*SELENOK*	DQ372075	F: CAGGAAACCCCCCTAGAAGAA
		R: CTCATCCACCGGCCATTG
*SELENOM*	FJ968780	F: CAGCTGAATCGCCTCAAAGAG
		R: GAGATGTTTCATGACCAGGTTGTG
*SELENON*	EF113595	F: ACCTGGTCCCTGGTGAAAGAG
		R: AGGCCAGCCAGCTTCTTGT
*SELENOO*	AK236851	F: CTTCCGACCCCAGATGGAT
		R: GGTTCGACTGTGCCAGCAT
*SELENOP*	EF113596	F: AACCAGAAGCGCCAGACACT
		R: TGCTGGCATATCTCAGTTCTCAGA
*SELENOS*	AY609646	F: GAGGCAGAGGCACCTGGAT
		R: CTGCTAAAGCCTCCTGTCGTTT
*SELENOT*	AY609428	F: GGCTTAATAATCGTTGGCAAAGA
		R: TGGCCCCATTGCCAGATA
*SELENOV*	GQ478346	F: CACTGGTCGCCAATGGATTC
		R: AGTGGCCAACGGAGAAAGC
*SELENOW*	NM_213977	F: CACCCCTGTCTCCCTGCAT
		R: GAGCAGGATCACCCCAAACA
*SEPHS2*	EF033624	F: TGGCTTGATGCACACGTTTAA
		R: TGCGAGTGTCCCAGAATGC
*TXNRD1*	AF537300	F: GATTTAACAAGCGGGTCATGGT
		R: CAACCTACATTCACACACGTTCCT
*TXNRD2*	GU181287	F: TCTTGAAAGGCGGAAAAGAGAT
		R: TCGGTCGCCCTCCAGTAG
*TXNRD3*	BX918808	F: GTGCCCTACGTTTATGCTGTTG
		R: TCCGAGCCACCAGCTTTG
*ACTB*	XM003124280	F: GGATGCAGAAGGAGATCACG
		R: ATCTGCTGGAAGGTGGACAG

*ACTB, beta actin; DIO, iodothyronine deiodinase; GPX, glutathione peroxidase; MSRB1, methionine sulfoxide reductase B1; SELENOF, H, I, K, M, N, O, P, S, T, V, and W, selenoproteins F, H, I, K, M, N, O, P, S, T, V, and W; SEPHS2, selenophosphate synthetase 2; and TXNRD, thioredoxin reductase*.

### Western Blot Analysis

Porcine mammary epithelial cells were seeded into six-well plates at 2 mL/well at 5 × 10^4^ cells/mL and cultured in a complete medium at 37°C and 5% CO_2_ for 48 h. Then, the cells were treated with different levels of Se-Met (0, 0.5, 1, 2, or 4 μM) for 48 h. After that, the cells were collected and homogenized in a RIPA lysis buffer (Beyotime, Nanjing, China). Western blot analysis was performed according to the procedures described in our previous study (26). The primary antibodies were as follows: (1) anti-SEPHS2 antibody (1:1,000, ab153878, Abcam, MA, USA), (2) anti-SELENOP antibody (1:1,000, sc-376858, Santa Cruz, CA, USA), (3) anti-GPX1 antibody (1:1,000, ab59546, Abcam, MA, USA), (4) anti-TXNRD1 antibody (1:1,000, ab78629, Abcam, MA, USA), and (5) anti-β-actin (1:1,000, bs-0061R, Bioss, Beijing, China).

### Antioxidant Enzymes Assay

Porcine mammary epithelial cells were seeded into six-well plates at 2 mL/well at 5 × 10^4^ cells/mL and cultured in a complete medium at 37°C and 5% CO_2_ for 48 h. Then, the cells were treated with different levels of Se-Met (0, 0.5, 1, 2, or 4 μM) for 48 h. After that, the cells were collected for glutathione peroxidase (GPX) activity and thioredoxin reductase (TRX) activity analysis. GPX activity (nmol NADPH/min/mL) was measured in the supernatant using a cellular glutathione peroxidase assay kit (Beyotime Institute of Biotechnology) that measures the coupled oxidation of NADPH during glutathione reductase (GR) recycling of oxidized glutathione from GPX-mediated reduction of t-butyl peroxide. For this assay, excess GR, glutathione, and NADPH were added according to the instructions of the manufacturer. The protein concentration of splenocyte lysate was measured using a Bicinchoninic Acid assay (Beyotime Institute of Biotechnology). Protein concentrations were used to correct the GPX activity of the cell lysates. GPX activity was expressed as mU/mg.

Thioredoxin reductase activity was measured using a thioredoxin reductase activity colorimetric assay kit (BioVision, USA). In this assay, TRX catalyzes the reduction of 5, 5′-dithiobis (2-nitrobenzoic) acid (DTNB) with NADPH to 5-thio-2-nitrobenzoic acid (TNB^2−^), which generates a strong yellow color (λ_max_ = 412 nm). Since other enzymes, such as glutathione reductase and glutathione peroxidase, can also reduce DTNB in crude biological samples, a TRX-specific inhibitor was utilized to determine the TRX-specific activity. Two assays were performed: the first measurement was the total DTNB reduction by the sample, and the second was the DTNB reduction by the sample in the presence of the TRX specific inhibitor. The difference between the two results represented the DTNB reduction by TRX.

### Statistical Analysis

Statistical analysis was conducted using SPSS 22.0 (SPSS, INC., Chicago, IL, USA). Data were analyzed using one-way ANOVA, followed by Duncan's multiple comparison test. The results are presented as mean and SEM. *p* < 0.05 was considered to be statistically significant. The figures and heatmap were drawn using Origin 8.0 software and Heatmap Illustrator software (HemI 1.0, version 1.0), respectively.

## Results

### Cell Viability

**Figure 1:**
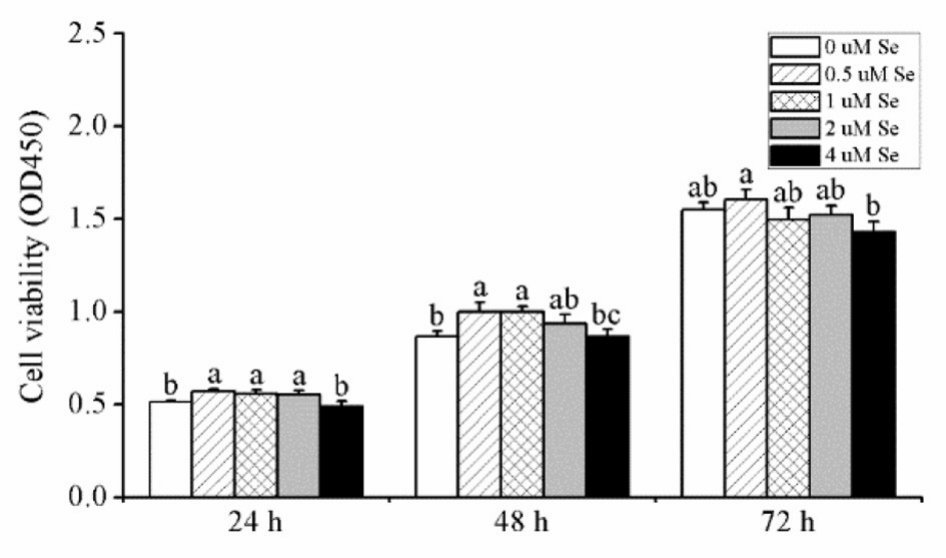
Effects of selenomethionine (Se-Met) supplementation on cell viability in porcine mammary epithelial cells (pMECs). The cells were incubated with different concentrations of Se-Met (0, 0.5, 1, 2, and 4 μM) for 24, 48, and 72 h. Cell viability was analyzed using the CCK-8 assay. The data are expressed as the mean ± SEM (*n* = 12). Different superscript letters indicate a significant difference (*p* < 0.05).

As shown in Figure 1, when pMECs were incubated for 24 h, compared with the control group,.5, 1., or 2.-μM Se-Met increased cell viability by 11.36, 8.59, and 8.06 (*p* < 0.05), respectively, while 4.μM Se-Met did not affect cell viability (*p* > 0.05). After 48 h of incubation, compared with the control group, 0.5- and 1.-μM Se-Met enhanced cell viability by 15.26 and 15.36% (*p* < 0.05), respectively, but 2.- or 4.-μM Se-Met did not influence cell viability (*p* > 0.05). When cells were treated for 72 h, Se-Met did not affect cell viability in comparison to the control group (*p* > 0.05), but 0.5-μM Se-Met improved cell viability compared with the 4.-μM Se-Met group by 12.55% (*p* < 0.05). Therefore, we selected 48 h as the incubation time for the subsequent experiments.

### Selenoprotein Transcriptome

**Figure 2:**
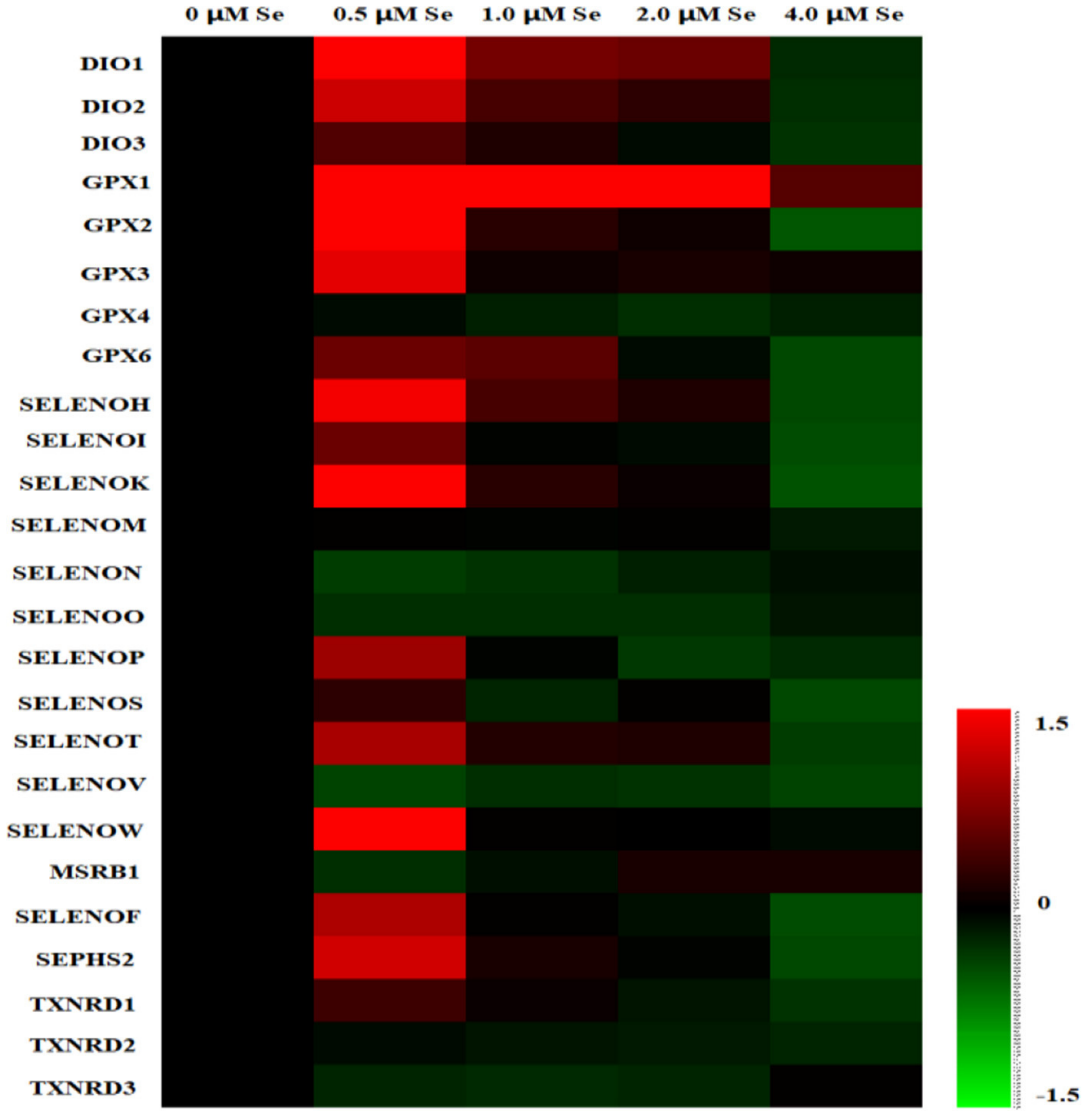
Heat map of the effects of selenomethionine (Se-Met) supplementation on the selenoprotein transcriptome in porcine mammary epithelial cells (pMECs). The cells were incubated for 48 h with different concentrations of Se-Met (0, 0.5, 1, 2, and 4 μM), and then collected for determination of mRNA expression. The heat map displays the extent of the changes. The color scale ranges from saturated red (1.5) to black (0) to saturated green (−1.5). Red and green colors represent increased and decreased expressions, respectively.

A heat map of the effects of Se-Met supplementation on the selenoprotein transcriptome of pMECs is shown in Figure 2. The results showed that the mRNA expression of most selenoproteins was first upregulated, and then gradually downregulated with increasing Se-Met concentration, peaking at 0.5-μM Se-Met.

**Figure 3:**
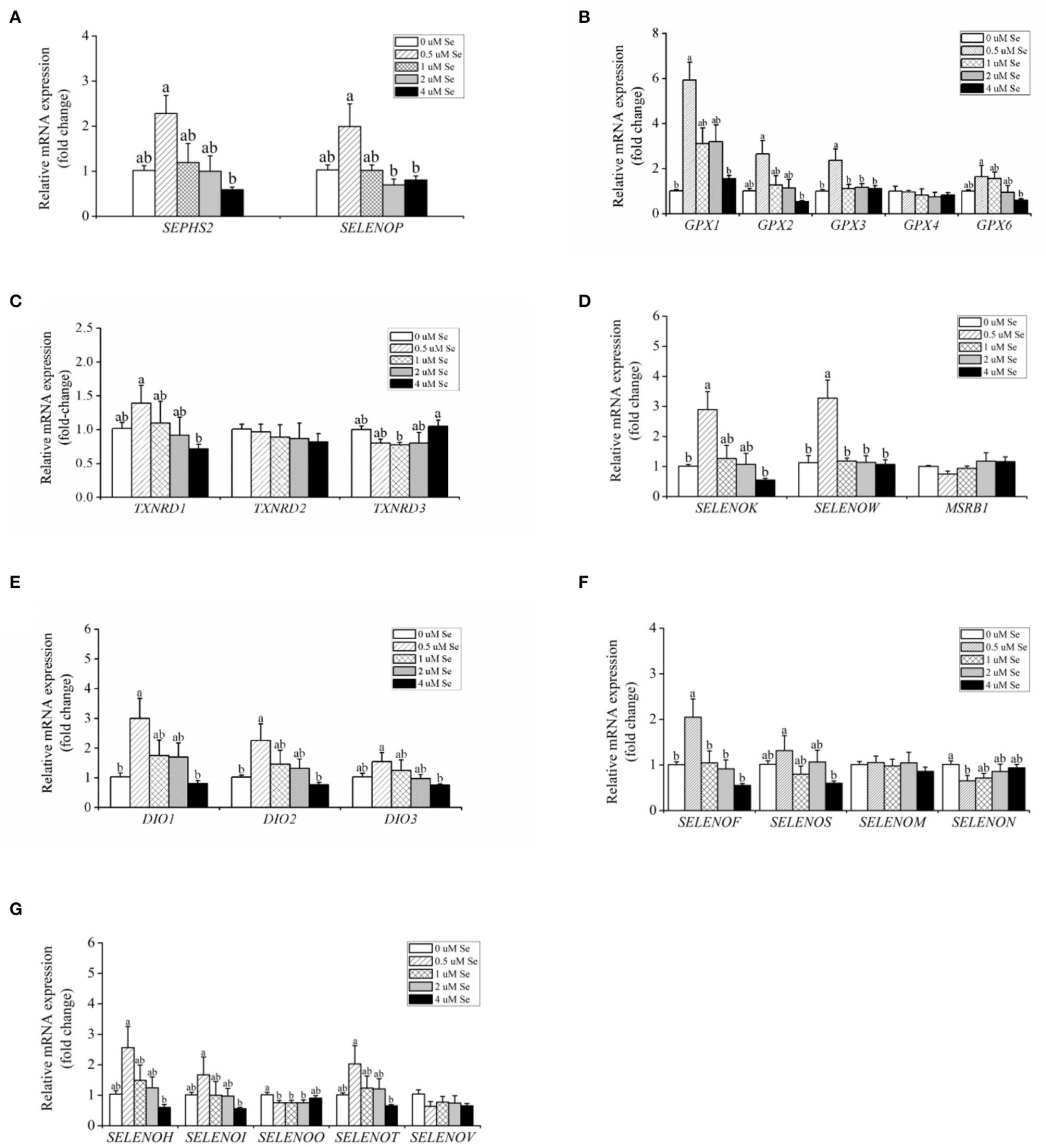
Effects of selenomethionine (Se-Met) supplementation on the relative mRNA expression of 25 selenoproteins in porcine mammary epithelial cells (pMECs). **(A)** selenoprotein *SEPHS2* and *SELENOP*; **(B)** the selenoprotein *GPX* family; **(C)** the selenoprotein *TXNRD* family; **(D)** antioxidant selenoproteins; **(E)** the selenoprotein *DIO* family; **(F)** protein-fold function selenoproteins; **(G)** other unknown functions of selenoproteins. The cells were incubated for 48 h with different concentrations of Se-Met (0, 0.5, 1, 2, and 4 μM) and then collected for determination of mRNA expression. The data are expressed as the mean ± SEM (*n* = 6). Different superscript letters indicate a significant difference (*p* < 0.05).

With increasing Se-Met concentration, the mRNA expression of *SEPHS2* and *SELENOP first* increased and then gradually decreased, reaching a maximum value at 0.5-μM Se-Met, which was higher than that of the 4.-μM Se-Met group (*p* < 0.05) (Figure 3A). For the *GPX* family, the mRNA expression of *GPX4* was unaffected by Se-Met treatments (*p* > 0.05). However, the mRNA expression of *GPX1, GPX2, GPX3*, and *GPX6 first* increased and then decreased with the increase of Se-Met concentration, reaching a plateau value at 0.5-μM Se-Met (Figure 3B). Regarding the *TXNRD* family, the mRNA expression of *TXNRD1 first* increased and then decreased, and the expression was the highest at 0.5-μM Se-Met, while the mRNA expression of *TXNRD3 first* increased and then decreased (*p* < 0.05) (Figure 3C). As presented in Figure 3D, Se-Met did not affect the mRNA expression of *MSRB1* (*p* > 0.05). As the Se-Met concentration increased, the mRNA expression of *SELENOK* and *SELENOW first* increased and then decreased, peaking at 0.5-μM Se-Met (*p* < 0.05). As displayed in Figure 3E, with the increasing Se-Met concentration, the mRNA expression of *DIO1, DIO2*, and *DIO3 first* increased and then decreased, reaching a maximum value at 0.5-μM Se-Met (*p* < 0.05). As shown in Figure 3F, Se-Met did not affect the mRNA expression of *SELENOM* (*p* > 0.05). With the increasing Se-Met concentrations, the mRNA expression of *SELENON first* increased and then increased, reaching a minimum value at 0.5-μM Se-Met. With the increasing Se-Met concentration, the mRNA expressions of *SELENOF* and *SELENOS* mRNA were increased firstly and then decreased, and the expression was the highest at 0.5-μM Se-Met (*p* < 0.05). As shown in Figure 3G, Se-Met did not affect the mRNA expression of *SELENOV* (*p* > 0.05). With the increasing Se-Met concentration, the mRNA expression of *SELENOO first* increased and then increased. With the increasing Se-Met concentration, the mRNA expression of *SELENOH, SELENOI*, and *SELENOT first* increased and then decreased, and the expression was the highest at 0.5-μM Se-Met. The mRNA expressions of *SELENOH, SELENOI*, and *SELENOT* were higher in the 0.5-μM Se-Met group than that in the 4.-μM Se-Met group (*p* < 0.05).

### Protein Expression of Selenoproteins

**Figure 4:**
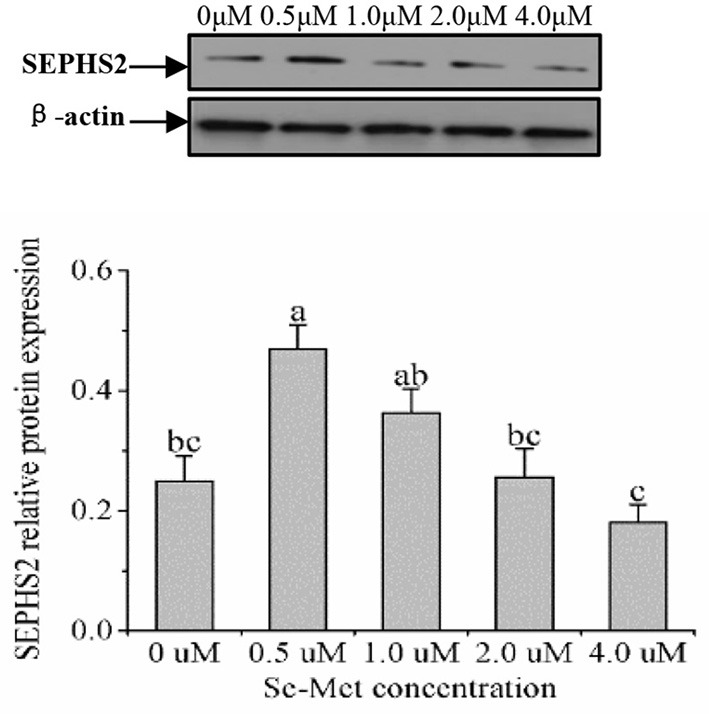
Effects of selenomethionine (Se-Met) supplementation on SEPHS2 protein expression in porcine mammary epithelial cells (pMECs). The cells were incubated for 48 h with different concentrations of Se-Met (0, 0.5, 1, 2, and 4 μM), and then collected for determination of protein expression. The data are expressed as the mean ± SEM (*n* = 3). Different superscript letters indicate a significant difference (*p* < 0.05).

**Figure 5:**
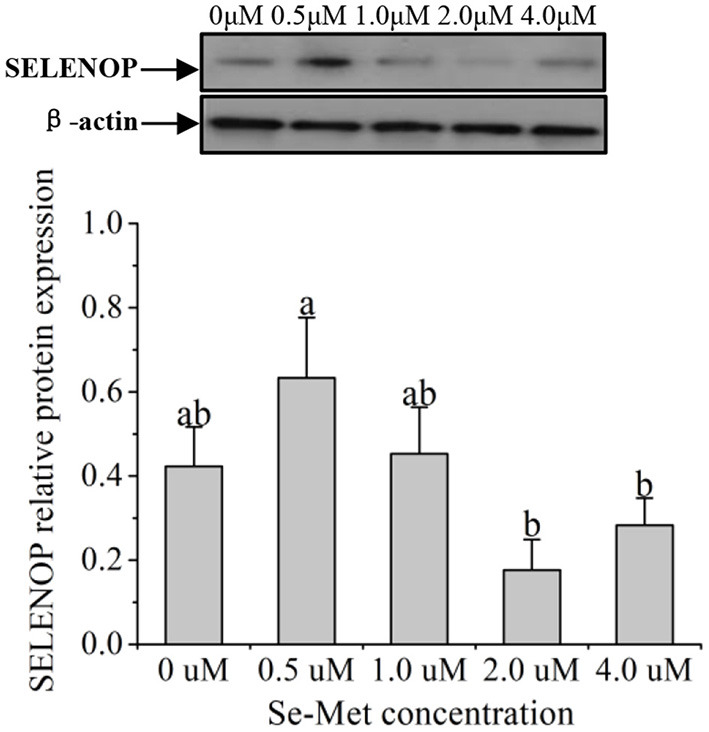
Effects of selenomethionine (Se-Met) supplementation on SELENOP protein expression in porcine mammary epithelial cells (pMECs). The cells were incubated for 48 h with different concentrations of Se-Met (0, 0.5, 1, 2, and 4 μM) and then collected for determination of protein expression. The data are expressed as the mean ± SEM (*n* = 3). The β-actin blot was reused as shown in Figure 4. Different superscript letters indicate a significant difference (*p* < 0.05).

**Figure 6:**
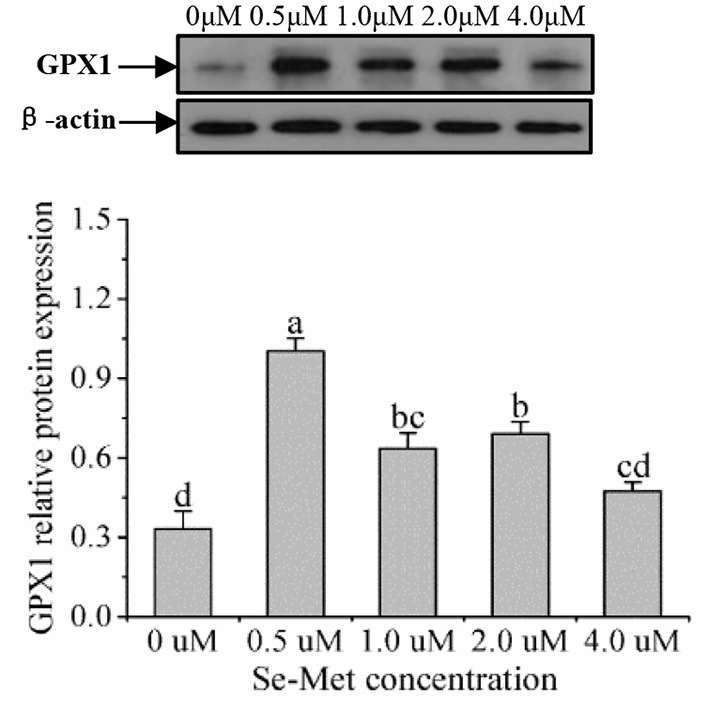
Effects of selenomethionine (Se-Met) supplementation on GPX1 protein expression in porcine mammary epithelial cells (pMECs). The cells were incubated for 48 h with different concentrations of Se-Met (0, 0.5, 1, 2, and 4 μM) and then collected for the determination of protein expression. The data are expressed as the mean ± SEM (*n* = 3). Different superscript letters indicate a significant difference (*p* < 0.05).

**Figure 7:**
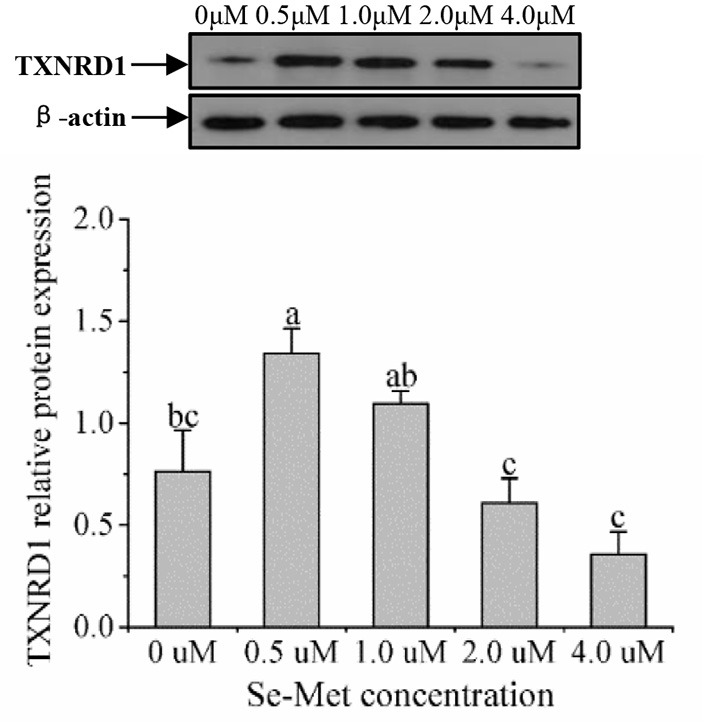
Effects of selenomethionine (Se-Met) supplementation on TXNRD1 protein expression in porcine mammary epithelial cells (pMECs). The cells were incubated for 48 h with different concentrations of Se-Met (0, 0.5, 1, 2, and 4 μM), and then collected for the determination of protein expression. The data are expressed as the mean ± SEM (*n* = 3). The β-actin blot was reused as shown in Figure 6. Different superscript letters indicate a significant difference (*p* < 0.05).

As displayed in Figure 4, the protein expression of SEPHS2 first increased and then decreased with the increasing Se-Met concentration, and the expression was the highest at 0.5-μM Se-Met. The protein expression of SEPHS2 was elevated when cells were treated with 0.5-μM Se-Met compared with the 0-, 2.-, 4.-μM Se-Met groups (*p* < 0.05). Additionally, the protein expression of SEPHS2 in the 1.-μM Se-Met group was increased compared with that in the 4.-μM Se-Met group (*p* < 0.05). As the Se-Met concentration increased, the protein expression of SELENOP first increased and then decreased; the expression was the highest at 0.5-μM Se-Met, and the expression of SELENOP in the 0.5-μM Se-Met group was higher than that in the 2.- and 4.-μM Se-Met groups (*p* < 0.05) (Figure 5). As presented in Figure 6, the protein expression of GPX1 first increased and then decreased with the increasing Se-Met concentration, peaking at 0.5-μM Se-Met. Additionally, the protein expression of GPX1 in the 0.5-μM Se-Met group was higher than that in the 0-, 1.-, 2.-, and 4.-μM Se-Met groups (*P* < 0.05), while the 1.- and 2.-μM Se-Met groups had higher GPX1 protein expression than the control group (*p* < 0.05). As represented in Figure 7, with the increasing Se-Met concentration, the protein expression of TXNRD1 increased first and then decreased; the expression was the highest at 0.5-μM Se-Met, and the expression of TXNRD1 in the 0.5-μM Se-Met group was higher than in the 0-, 2.-, and 4.-μM Se-Met groups (*p* < 0.05), while the 1.-μM Se-Met group was higher than that in the 2.- and 4.-μM Se-Met groups (*p* < 0.05). The original images for the blots are provided in the Supplemental Materials.

### GPX and TRX Activities

**Figure 8:**
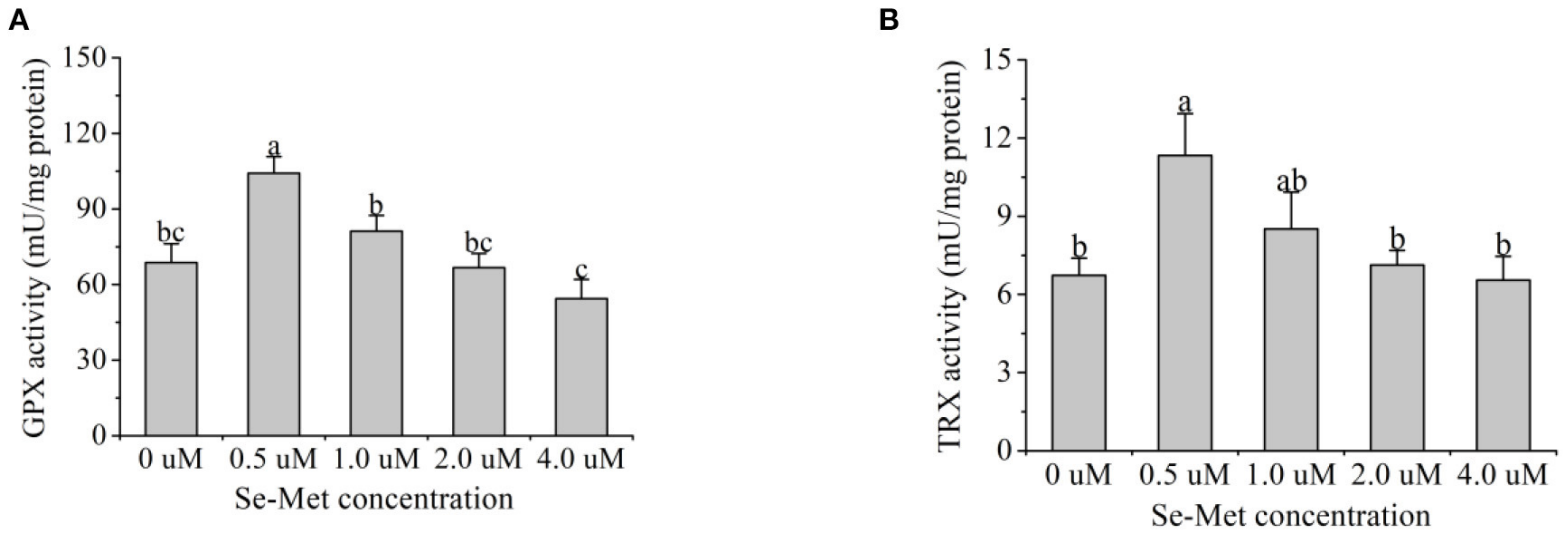
Effects of selenomethionine (Se-Met) supplementation on glutathione peroxidase (GPX) **(A)** and thioredoxin reductase (TRX) activity **(B)** in porcine mammary epithelial cells (pMECs). The cells were incubated for 48 h with different concentrations of Se-Met (0, 0.5, 1, 2, and 4 μM), and then collected for GPX and TRX activity analysis. The data are expressed as the mean ± SEM (*n* = 6). Different superscript letters indicate a significant difference (*p* < 0.05).

As presented in Figure 8, with the increasing Se-Met concentrations, GPX activity first increased and then decreased, and the activity was highest at 0.5-μM Se-Met. The GPX activity in the 0.5-μM Se-Met group was higher than that in the 0-, 2.-, and 4.-μM Se-Met group (*p* < 0.05), while GPX activity in the 1.-μM Se-Met group was elevated compared with that in the 4.-μM Se-Met group (*p* < 0.05). With the increasing Se-Met concentration, TRX activity first increased and then decreased, peaking at 0.5 μM. The TRX activity in the 0.5-μM Se-Met group was higher than that in the 0-, 1.-, 2.-, and 4.-μM Se-Met groups (*p* < 0.05).

## Discussion

This study was conducted to investigate the effects of Se-Met on selenoprotein expression and antioxidant function in pMECs to reveal the underlying molecular mechanism of Se-Met on the lactation performance and antioxidant capacity of sows *in vitro*. Yan et al. (31) found that Se promotes the proliferation of chondrocyte ATDC5 cells by increasing intracellular ATP content. Hao et al. (32) treated primary porcine splenocytes with 0-,.5-, 1-, 2-, 4-, 8-, and 16-μM selenite or Se-Met and found that T-cell proliferation gradually increases in response to Se levels, with the maximum value at 2-μM Se-Met or sodium selenite. Similarly, Zhuang et al. (33) treated primary porcine splenocytes with 0-,.5-, 2-, and 5-μM sodium selenite, and found that cell proliferation gradually increases with increasing Se levels and reaches a maximum value at 2-μM sodium selenite. The results of this experiment showed that, with increasing Se-Met levels, cell viability first increased and then decreased, with a maximum value at 0.5-μM Se-Met. Our results suggest that normal levels of Se can promote cell growth, while supra-nutritional levels of Se inhibit cell proliferation (21).

In the present study, the expression of SEPHS2 at either the mRNA or protein level was highest at 0.5-μM Se-Met, which indicates selenoprotein biosynthesis was most favorable at 0.5-μM Se-Met. It has been reported that selenoprotein P (SELENOP) is the primary transporter of Se in milk (34). The Se content in the milk of female mice with a knockout of the *SELENOP* gene is reduced by 73%, and the Se intake of suckling rats is reduced by 65% (34). In other words, a knockout of the *SELENOP* gene reduces Se transfer from lactating mothers to suckling offsprings (34). Hill et al. (34) also reported that milk SELENOP is synthesized by the mammary gland. In the present experiment, the expression of SELENOP at either the mRNA or protein level was the highest at 0.5-μM Se-Met, which indicates that Se transfer was most favorable at 0.5-μM Se-Met. Therefore, selenoprotein synthesis (SEPHS2) and transport (SELENOP) were highest at 0.5-μM Se-Met, which makes it easy to understand that most other selenoproteins were upregulated at 0.5-μM Se-Met.

In this study, as the Se-Met concentration increased, the mRNA expression of most selenoproteins, including *SEPHS2, SELENOP, GPX1, GPX2, GPX3, GPX6, TXNRD1, SELENOK, SELENOW, DIO1, DIO2, DIO3, SELENOF, SELENOS, SELENOH, SELENOI*, and *SELENOT*, was first increased and then decreased, reaching a maximum at 0.5-μM Se-Met. It has been reported that Se can affect the selenoprotein transcriptome in mouse ATDC5 chondrocytes and human C28/I2 cells (35). Se supplementation was shown to significantly upregulate the mRNA expression of *GPX1, SELENOH, SELENON, SELENOP*, and *SELENOW* in ATDC5 cells and *GPX1, SELENOH, SELENON, SELENOP, SELENOW*, and *GPX3* in C28/I2 cells and significantly downregulate the mRNA expression of *SEPHS2* and *SELENOO* in ATDC5 cells and *SEPHS2, SELENOO*, and *TXNRD2* in C28/I2 cells (35). *In vivo* experiments also demonstrated that the selenoprotein transcriptome in the liver and muscle of chickens is regulated by different Se sources in the diet (36). Se has been reported to regulate the selenoprotein transcriptome of chicken embryonic neurons, and the mRNA expression of *SELENOT, SELENOF, SELENOU, GPX3, SELENOK, SELENOW, GPX4, SELENOP*, and *GPX2* is sensitive to Se levels in the diet (37). Huang et al. (38) found that the Se-deficiency disease exudative diathesis of chickens is related to the downregulation of seven common selenoprotein genes, including *GPX1, GPX4, SELENOW, SELENON, SELENOP, SELENOO*, and *SELENOK*, in the liver and muscle. However, Zhou et al. (39) found that the expression of selenoprotein genes in the thyroid and pituitary of weaned piglets is unaffected by a deficiency or excess of Se in the diet. Therefore, as reported by Liu et al. (40), Se supplementation does not globally regulate all selenoproteins, and the expression situation is also different due to different tissues. Miranda et al. (41) reported that Se-Met promotes the expression of GPX1 and GPX3 in primary bovine mammary epithelial cells. Hao et al. (32) also found that Se-Met promoted the mRNA and protein expression of GPX1 and SELENOS without affecting *GPX4* mRNA expression in primary porcine splenocytes, which is consistent with our results. However, Se-Met does not alleviate the toxic effects of aflatoxin B1 on primary porcine spleen cells treated with GPX1-siRNA and SELENOS-siRNA (32, 33). The results of Hao et al. (32) suggested that Se-Met exerts biological functions by regulating the expression of GPX1 and SELENOS. Does Se deficiency reduce GPX activity of cells? In primary cultured pig thyrocytes, hydrogen peroxide causes a decrease in GPX activity and activation of caspase-3, and Se deficiency aggravates cell apoptosis due to decreased GPX activity (42). Chen et al. (43) found that oxidative stress induces the reproduction of porcine circovirus PCV2, while 6-μM Se-Met inhibits the proliferation of PCV2. However, Se-Met did not alleviate the proliferation of PCV2 treated with GPX1-siRNA (43), suggesting that GPX1 may be a critical factor blocking oxidative stress and porcine circovirus reproduction (44). The results of this experiment showed that the mRNA expression of *GPX1, GPX2, GPX3*, and *GPX6* was highest at 0.5-μM Se-Met. Western blot results also showed that GPX1 protein expression was highest at 05-μM Se-Met, indicating that 0.5-μM Se-Met is most beneficial for the synthesis of GPX1.

In the present study, the mRNA and protein expression of TXNRD1 was highest at 0.5-μM Se-Met. Studies have shown that thioredoxin reductase deficiency exacerbates oxidative stress, mitochondrial disorders, and cell death in N27 cells (45). Se upregulates the endogenous antioxidant system of human placental trophoblast cells (Bewo and Jeg-3 cells), thereby protecting cells from oxidative damage (46, 47). However, Se did not relieve cellular oxidative stress after cells were treated with auranofin (a specific blocker of GPX and TRX), suggesting that GPX and TRX are two crucial members of alleviating oxidative stress (46, 47). Se plays a vital role in the antioxidant system of animal organisms. The results of this experiment showed that the activities of GPX and TRX were first increased and then decreased with increasing Se-Met concentration, reaching a maximum value at 0.5-μM Se-Met. Miranda et al. (48) found that Se-Met increases GPX activity in bovine mammary epithelial cells and restores intracellular peroxide to normal levels. *In vivo* experiments also found that Se treatment can block cadmium-induced reactive oxygen species (ROS) production in mice, inhibit cadmium-induced mitochondrial membrane collapse, prevent cytochrome C release, and inhibit caspase death receptor activation (49). Higuchi et al. (50) reported that dry eye disease is thought to be a disease induced by oxidative stress and that Se protects the oxidative stress of the corneal epithelium. Although a lot of studies have reported the beneficial effects of Se-Met or yeast Se in dairy animals during the lactation or perinatal period, to the best of our knowledge, the present study is the first to investigate the impact of Se-Met on pMECs. The current experiment provides insights into the key regulatory role of Se-Met in the selenoprotein transcriptome of pMECs while revealing its importance for improving mammary gland health in sows. However, further studies are required to explore the regulatory effects of Se-Met on the synthesis and secretion of milk components, including milk fat (fatty acids), protein (amino acids), and lactose using pMECs and animal models.

## Conclusions

In conclusion, 0.5-μM Se-Met promotes cell viability partially by improving selenoprotein expression and antioxidant function in pMECs. Our results provide evidence for the potential ability of Se-Met for improving mammary gland health in sows.

## Data Availability Statement

The datasets generated for this study can be found in online repositories. The names of the repository/repositories and accession number(s) can be found in the article/Supplementary Material.

## Author Contributions

WG and SZ: conceptualization. FC: methodology and supervision. JY: software. YZ and MT: validation. YZ: formal analysis. JC: investigation and writing—original draft preparation. WG: resources, writing—review and editing, project administration, and funding acquisition. YL: data curation. SZ: visualization. All authors have read and agreed to the published version of the manuscript.

## Conflict of Interest

The authors declare that the research was conducted in the absence of any commercial or financial relationships that could be construed as a potential conflict of interest.

## Publisher's Note

All claims expressed in this article are solely those of the authors and do not necessarily represent those of their affiliated organizations, or those of the publisher, the editors and the reviewers. Any product that may be evaluated in this article, or claim that may be made by its manufacturer, is not guaranteed or endorsed by the publisher.
